# The effectiveness of manual therapy on pain, physical function, and nerve conduction studies in carpal tunnel syndrome patients: a systematic review and meta-analysis

**DOI:** 10.1007/s00264-021-05272-2

**Published:** 2021-12-03

**Authors:** Sandra Jiménez-del-Barrio, Aida Cadellans-Arróniz, Luis Ceballos-Laita, Elena Estébanez-de-Miguel, Carles López-de-Celis, Elena Bueno-Gracia, Albert Pérez-Bellmunt

**Affiliations:** 1 Clinical Research in Health Sciences Group. Department of Surgery, Ophthalmology, Otorhinolaryngology, and Physiotherapy, Faculty of Health Sciences, University of Valladolid, Calle Universidad S/N, 42002 Soria, Spain; 2 Physiotherapy Department, Faculty of Medicine and Health Sciences, International University of Catalunya, Universitat Internacional de Catalunya, 08195 Sant Cugat del Vallès, Barcelona Spain; 3 Department of Physiatrist and Nursery, Faculty of Health Sciences, University of Zaragoza, Calle Domingo Miral S/N, 50009 Zaragoza, Spain; 4 Jordi Gol I Gurina University Institute for Research in Primary Health Care Foundation, Barcelona, Spain

**Keywords:** Carpal tunnel syndrome, Manual therapy, Median nerve, Neuropathies, Meta-analysis

## Abstract

**Aim of the study:**

Systematic review and meta-analysis to assess the effectiveness of manual therapy in improving carpal tunnel syndrome (CTS) symptoms, physical function, and nerve conduction studies.

**Method:**

MEDLINE, Web of Science, SCOPUS, Cochrane Library, TRIP database, and PEDro databases were searched from the inception to September 2021. PICO search strategy was used to identify randomized controlled trials applying manual therapy on patients with CTS. Eligible studies and data extraction were conducted independently by two reviewers. Methodology quality and risk of bias were assessed by PEDro scale. Outcomes assessed were pain intensity, physical function, and nerve conduction studies.

**Results:**

Eighty-one potential studies were identified and six studies involving 401 patients were finally included. Pain intensity immediately after treatment showed a pooled standard mean difference (SMD) of − 2.13 with 95% confidence interval (CI) (− 2.39, − 1.86). Physical function with Boston Carpal Tunnel Syndrome Questionnaire (BCTS-Q) showed a pooled SMD of − 1.67 with 95% CI (− 1.92, − 1.43) on symptoms severity, and a SMD of − 0.89 with 95% CI (− 1.08, − 0.70) on functional status. Nerve conduction studies showed a SMD of − 0.19 with 95% CI (− 0.40, − 0.02) on motor conduction and a SMD of − 1.15 with 95% CI (− 1.36, − 0.93) on sensory conduction.

**Conclusions:**

This study highlights the effectiveness of manual therapy techniques based on soft tissue and neurodynamic mobilizations, in isolation, on pain, physical function, and nerve conduction studies in patients with CTS.

**Supplementary Information:**

The online version contains supplementary material available at 10.1007/s00264-021-05272-2.

## Introduction

Carpal tunnel syndrome (CTS) is considered the result of the compression of the median nerve in the carpal tunnel [1, 2] and is one of the most common upper extremity neuropathies [3–5]. Recent studies show that CTS’s prevalence and the incidence are increasing in the last years [6, 7], causing important socioeconomic cost [4]. Patients with CTS often report pain, paraesthesia, sensory disturbances, weakness in the hand and wrist, causing a physical function decrease that affects daily living activities [8, 9]. Due to the high prevalence of CTS, its effects on daily living activities and the health care cost are necessary to identify the best therapeutic approaches [4]. Secondary causes have been described of CTS including traumatism, metabolic conditions, infections, neuropathies, or other systemic disorders. However, most of cases of CTS are idiopathic [4, 5].

Clinical guidelines recommend conservative treatment to manage symptoms and loss of function of patients with mild to moderate CTS [10]. The leading conservative treatments are splinting, steroid injection, electrotherapy, and manual therapy [11, 12]. Manual therapy applied on CTS patients includes different interventions such as manual and instrumental soft tissue mobilizations, massage therapy, bone mobilizations or manipulations, and neurodynamic techniques, focused on skeletal system or soft tissue [13]. As previous studies suggested, when the CTS has not a clear cause, the manual therapy applications could reduce the epineural tethering in the forearm and could improve the nerve gliding in the carpal tunnel during the movement of the wrist, fingers, or elbow. The number of studies analyzing manual therapy interventions has increased in last years, and they have shown positive effects on symptoms and physical function in patients with CTS [14–20]. Although a recent review has assessed the effects of conservative treatments in patients with CTS [21], to the best of our knowledge, no systematic review with meta-analysis has been performed in order to assess the effectiveness of manual therapy on the main symptoms, function, and nerve conduction studies in patients with CTS [22, 23].

The aim of this systematic review and meta-analysis was to assess the effectiveness of manual therapy qualitatively and quantitatively in improving CTS symptoms such as pain, physical function, and nerve conduction studies.

## Methods

A systematic review of the scientific literature according to the Preferred Reporting Items for Systematic Reviews and Meta-Analysis (PRISMA) statement checklist and the Guidelines of Cochrane Handbook for Systematic Reviews of Interventions Version 6 was conducted [24]. The study was registered in the PROSPERO with the following registration number CRD42020167559.

The PICO strategy was developed in order to perform an accurate search strategy. Population were patients diagnosed with CTS; intervention studied was manual therapy techniques applied in isolation; comparison was control, placebo, sham, or simulated intervention; main outcomes were pain intensity, functionality, disability, and nerve conduction studies. Keywords used to develop the search strategy are shown on Table 1.

**Table 1:** Keywords used for the search strategy

Population	Intervention	Control	Outcomes
Carpal tunnel syndrome	Manual therapy Neurodynamic Neural mobilization Graston Neural tension Mobilization Manipulation Massage Fibrolysis Diacutaneous Surgery Surgical Resease	Control Placebo Sham Simulated	Symptom* Functi* Nerve conduction studies Functional capacity Disability Ability Pain

MEDLINE, Web of Science, SCOPUS, Cochrane Library, TRIP database, and PEDro were the databases used for the computerized search strategy. The last search was performed on September 1, 2021. The strategy was modified and adapted for each searched database with no restriction of language. Reference list of the included studies and the relevant reviews were also manually screened to identify additional studies for inclusion. Search strategies used are available in Appendix 1.

Studies were eligible if they met the following criteria: (1) randomized controlled trial design, (2) patients diagnosed with CTS, (3) manual therapy techniques applied in isolation, (4) compared to control, sham, simulated or placebo intervention, (5) studies measuring pain intensity, functionality, disability, and nerve conduction studies. Studies were excluded if any of the following criteria were met: (1) case reports, non-randomized controlled trials, reviews, crossover trial, (2) the procedure of the intervention was unspecified, (3) the treatment consisted of surgical procedures, (4) numerical data results were not provided. Two independent reviewers selected the studies by reading the title, abstract, and full texts. Any discrepancies were solved by a third independent reviewer.

Data collected for studies included in the present review was used to describe the study characteristics table (Table 2). Data extracted were the following: (1) author’s last name (2) year; (3) study design; (4) sample size, gender, and mean age; (5) pathology; (6) control group intervention; (7) experimental group intervention; (8) outcome measures and tool used; (9) main results.

**Table 2:** Study characteristics

Population						Intervention					
Author (year)	Study design	*N*	Gender	Mean age	Pathology	Control/placebo/sham group	Intervention group	Comparative intervention group	Outcomes (tool)	Follow-up	Main results
Wolny et al. 2019 [27]	RCT	103 (58/45)	11/92	54.6 (9.1)/53.1 (10.1)	CTS	No intervention	NDS Three series of 60 repetitions of sliding and tensioning neurodynamic techniques separated by inter-series intervals of 15 s 2/weeks (total: 20 sessions/10 weeks)		Pain intensity (NPRS) Function (BCTS-Q SSS) Function (BCTS-Q FSS) Nerve conduction (SCV) Nerve conduction (MCV) Nerve conduction (MT)	Before and after technique. (10 weeks)	↑ Pain, function, and nerve conduction
Wolny et al. 2018 [25]	RCT	150 (78/72)	15/135	54.2 (9.48)/52.2 (10.4)	CTS	Placebo therapy 3 series of 60 repetitions of placebo glide and tension neurodynamic techniques separated by interseries intervals of 15 s 2/week for 20 sessions (20 sessions/10 weeks)	NDS 3 series of 60 repetitions of glide and tension neurodynamic techniques separated by interseries intervals of 15 s 2/week (total: 20 sessions/10 weeks)		Pain intensity (NPRS) Function (BCTS-Q SSS) Function (BCTS-Q FSS) Nerve conduction (SCV) Nerve conduction (MCV) Nerve conduction (MT)	Before and after technique. (10 weeks)	↑ Pain, function, and nerve conduction
Jiménez-del-Barrio et al. 2018 [15]	RCT	52 (24/27)	11/41	44.97 (9.34)/48.83 (7.98)	CTS	Sham therapy 5 sessions All intervention lasted an average of 17.77 days (SD: 0.8) with an interval of two to five days between each session	DF 5 sessions All intervention lasted an average of 17.77 days (SD: 0.8) with an interval of two to five days between each session		Pain intensity (VAS) Nerve conduction (DML) Nerve conduction (SCV)	Before and after treatment, and one moth-follow-up	Posttreatment: ↑ pain and nerve conduction one month follow-up: ↑ pain
Jiménez-del-Barrio et al. 2021 [26]	RCT	39 (18/21)	5/34	45.5 (9.6)/49.6 (7.6)	CTS	Sham therapy 5 sessions All intervention lasted an average of 17.77 days (SD: 0.8) with an interval of two to five days between each session	DF 5 sessions All intervention lasted an average of 17.77 days (SD: 0.8) with an interval of two to five days between each session		Pain intensity (VAS) disability (BCTS-Q) nerve conduction (SCV)	Before and after treatment	↑ VAS nad nerve conduction
	RCT	21 (7/7/7)	0/21	47.1 (S.D. 14.8)	CTS	No intervention	Neurodynamic mobilization ULTT2a mobilization	Bone mobilization carpal bone mobilization (posterior-anterior and/or anterior–posterior mobilization techniques) and flexor retinaculum stretch	Pain intensity (VAS) Function (PRS) Function (FBS)	Before and after treatment	↑ Pain and function
Shem etal. 2020 [28]	RCT	36 (17/19)	10/26	48.18 (7.18)/50.05 (9.71)	CTS	Sham therapy 30 s at a time, four times a day for six weeks	Self-myofascial stretching of the carpal ligament 30 s at a time, four times a day for six weeks		Pain intensity (VAS) Function (BCTS-Q FSS) Function (BCTS-Q SSS) Nerve conduction (sensory DL) Nerve conduction (motor DL)	Before and after treatment	↑ Pain and nerve conduction

*RCT*, randomized controlled trial; *CTS*, carpal tunnel syndrome; *NDS*, neurodynamic mobilizations; *DF*, diacutaneous fibrolysis; *VAS*, visual analog scale; *DL*, distal latency; *SCV*, sensory conduction velocity; *MCV*, motor conduction velocity; *DL*, distal latency; *BCTS-Q*, Boston Carpal Tunnel Syndrome Questionnaire

In order to assess the methodology quality and risk of bias of studies included in this systematic review, Physiotherapy Evidence Database (PEDro) scale was used (Table 3). It was assessed independently by two authors and a third author intervened in case of disagreement. The PEDro scale is an 11-item scale that relates the external validity, and the internal validity of a study. One point is awarded if the criteria is clearly satisfied as assessed by following cut-points 9–10: excellent; 6–8: good; 4–5: fair; < 4: poor.

**Table 3:** PEDro scale

PEDro score	Item 1	Item 2	Item 3	Item 4	Item 5	Item 6	Item 7	Item 8	Item 9	Item 10	Item 11	Total
Jiménez et al. 2018 [18]	Yes	Yes	Yes	Yes	Yes	No	Yes	Yes	No	Yes	Yes	9
Jiménez et al. 2021 [26]	Yes	Yes	Yes	Yes	Yes	No	Yes	Yes	Yes	Yes	Yes	10
Wolny et al. 2018 [25]	Yes	Yes	Yes	Yes	Yes	No	Yes	Yes	No	Yes	Yes	9
Wolny et al. 2019 [27]	Yes	Yes	Yes	Yes	Yes	No	Yes	Yes	No	Yes	Yes	9
Tal-Akabi et al. 2000 [14]	Yes	No	No	Yes	No	No	Yes	Yes	Yes	Yes	No	6
Shem et al. 2020 [28]	Yes	Yes	Yes	Yes	Yes	Yes	Yes	No	No	Yes	Yes	9

RevMan 5.3 software package was used to develop all statistical analysis based on mean scores and standard deviation. Intervention effects were assessed by introducing changes between the baseline and the post-intervention assessment, comparing manual therapy group versus control group, provided on each study. If no post-intervention mean differences and standard deviation were provided by the authors, it was calculated by SPPS.

Standard mean difference (SMD) effect was used for all continuous outcomes because different scales and units were used in the main outcomes assessed. Random effects were used and the heterogeneity was assessed visually by means of forest plots and by reporting the *I*^2^ statistic (low, moderate, or high if *I*^2^ statistic was < 25%, 25–75%, or > 75% respectively). Pooled SMD and 95% confidence interval were calculated. If heterogeneity is considered significant > 70 *I*^2^, sensitivity analysis was conducted. Funnel plots were used to illustrate the risk of publication bias.

## Results

**Fig. 1:**
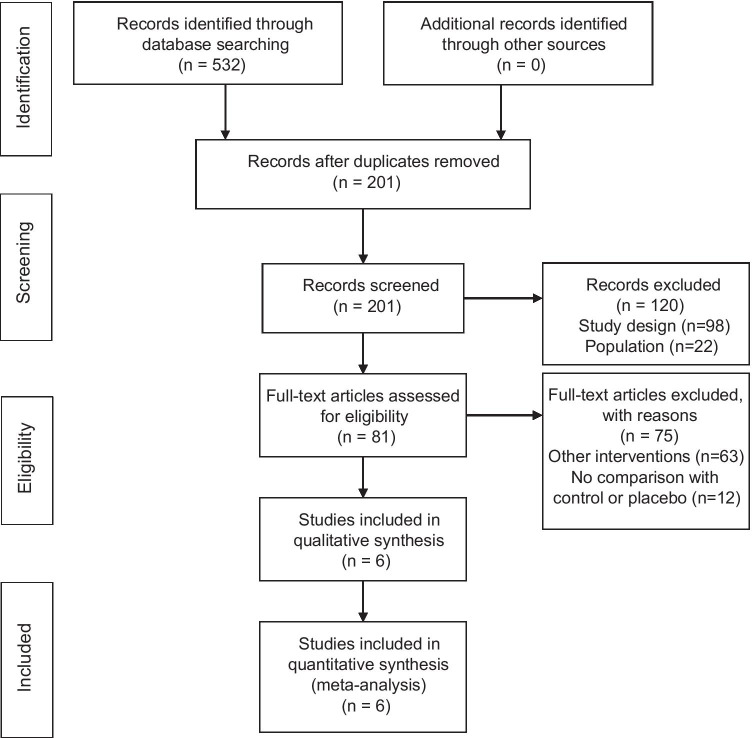
PRISMA flow diagram

The search strategy generated a total of 532 studies that were potentially eligible for this review. Analysis of Cohen’s Kappa index showed a *k* = 0.48 categorized as moderate agreement. Finally, six studies were included in the qualitative and quantitative synthesis. Figure 1 shows the PRISMA flowchart with the study selection procedure.

Characteristics of the studies included in this systematic review and meta-analysis are shown in Table 2. Studies involved 401 patients (52 males and 349 females) with CTS mean age ranged from 44.97 to 54.2 years. Three studies applied neurodynamic mobilizations based on sliding and tensioning neurodynamic techniques, two studies applied the diacutaneous fibrolysis technique and one study applied a myofascial stretching approach.

All studies included in this systematic review and meta-analysis measured pain intensity. Four studies considered function and five assessed nerve conduction.

Other outcomes measured in the studies but not related to this systematic review were grip pinch, range of movement or upper limb tension test.

The methodological quality assessed by PEDro scale indicated an overall high quality of the studies included in this systematic review. Five of the six studies scored between 8 and 11 with an average of 8.6 [15, 25–28]. Only one study scored a lower score of 6/11 on the scale [14]. The principal bias found between all studies was that there was not blinding of therapist who administered the therapy. However, due to the nature of the manual therapy techniques, it is not possible to completely blind therapist. Another common feature found was that results were not presented for all subjects initially included, due to the follow-up loses. Furthermore, in those cases, the data were not analyzed on an “intention to treat” basis.

Six studies were included on the quantitative synthesis. Pain, function, and nerve conduction outcomes were tested under the manual therapy versus a control therapy comparison for this meta-analysis. Only the immediate effects after technique application could be evaluated.

The study by Tel-Akabi et al. (2000) did not provide data for standard deviation but provided data for all patients (*n* = 7), so calculation could be performed.

### Pain

**Fig. 2:**
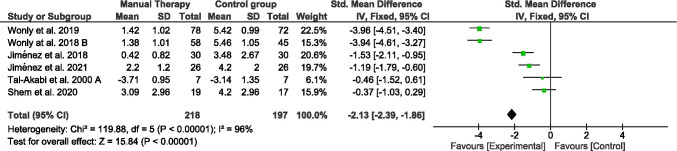
Forest plot of comparison. Manual therapy vs control group. Outcome: pain

The pain intensity effects immediately after manual therapy techniques were tested in all studies included (Fig. 2). Two hundred eighteen participants were included in the manual therapy groups and a hundred ninety-seven in the control group. Four of the five studies included used the visual analog scale (mm) for the pain assessment [14, 15, 26, 28], whereas the two other used the pain rating scale (from 0 to 10) [25, 27]. Pain intensity showed a pooled SMD (95% CI) of − 2.13 (− 2.39, − 1.86). Heterogeneity analysis by *I*^2^ characteristics showed a high heterogeneity (96%). To detect whether any of the studies might have a greater influence on the heterogeneity results, a sensitivity test was performed by repeating the meta-analysis excluding one study at a time. We observed that removing any study heterogeneity and results did not notably decrease.

### Function

**Fig. 3:**
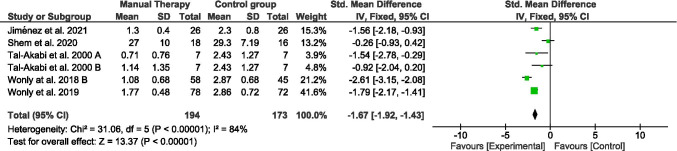
Forest plot of comparison. Manual therapy vs control group. Outcome: symptom severity scale (BCTS-Q)

Function outcome was assessed by means of the Boston Carpal Tunnel Syndrome Questionnaire (BCTS-Q) in all the studies included for this meta-analysis. This scale is sub-divided into two dimensions. One dimension focuses on the implication of symptom severity on functional tasks (Symptom Severity Scale), involving 11 items (Fig. 3), and the other one on function status properly (Functional Status Scale), involving 8 items (Fig. 5). However, not all studies provided data for both dimensions. Thus, the meta-analysis was conducted separately for each sub-scale.

All studies assessed function by means of symptom severity scale (BCTS) immediately after treatment [14, 15, 25, 27, 28]. A hundred ninety-four participants were involved in the manual therapy groups and a hundred seventy-three in the control group. Function changes showed a pooled SMD (95% CI) of − 1.67 (− 1.92, − 1.43). Analysis by *I*^2^ characteristics showed a high heterogeneity (84%) sensitivity analysis showed that removing Shem et al. (2020) [28] study may decrease the heterogeneity to moderate, which indicates. However, the SMD did not notably change after repeating the meta-analysis without this study.

On the other hand, four of the studies included in this meta-analysis provided data about functional status scale of BCTS [14, 25, 28]. Two hundred ninety-three participants were included in the manual therapy groups and two hundred and thirty-eight in the control group. Analysis showed a pooled SMD (95% CI) of − 0.89 (− 1.08, − 0.70). Heterogeneity analysis by *I*^2^ characteristics showed a high heterogeneity (94%). Removing any study for sensibility analysis, heterogeneity and results did not notably change.

### Nerve motor conduction

The nerve motor conduction was tested immediately after treatment in four studies included in this systematic review (Fig. 5). A hundred eighty-five participants were involved in the manual therapy group and a hundred sixty-four in the control group. Four studies provided data of nerve motor conduction by nerve conduction studies, two obtained latencies [15, 28] and two motor conduction velocity and distal motor latency [25, 27] by nerve conduction studies. Nerve conduction showed a pooled SMD (95% CI) of − 0.19 (− 0.40, − 0.02). Heterogeneity analysis by *I*^2^ characteristics showed a moderate heterogeneity (69%). Removing Jiménez et al. (2018) [15] study, for the sensitivity analysis showed that *I*^2^ drops to 0%, which may indicate that without this study, the homogeneity would be almost perfect. However, when repeating the meta-analysis without it, the results were not notably modified.

### Nerve sensory conduction

The nerve sensory conduction was assessed in five studies included in this systematic review (Fig. 6). Two hundred eleven participants were part of the manual therapy group and a hundred ninety of the control group. Five studies provided data of sensory conduction velocity by nerve conduction studies [15, 25–28]. Nerve conduction showed a pooled SMD (95% CI) of − 1.15 (− 1.36, − 0.93). Moderate heterogeneity was observed in *I*^2^ (75%). The sensitivity analysis indicated that the study of Shem et al. (2020) significantly contributed to this value because the heterogeneity dropped to 0% when was excluded. Likewise, the results did not significantly change.

**Fig. 4:**
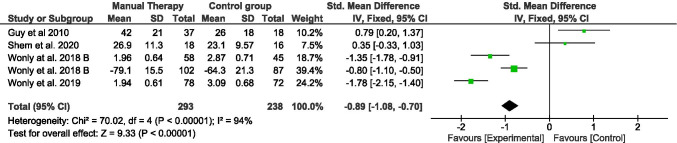
Forest plot of comparison. Manual therapy vs control group. Outcome: functional status scale (BCTS-Q)

**Fig. 5:**
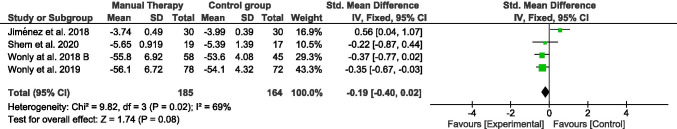
Forest plot of comparison. Manual therapy vs control group. Outcome: nerve motor conduction

**Fig. 6:**
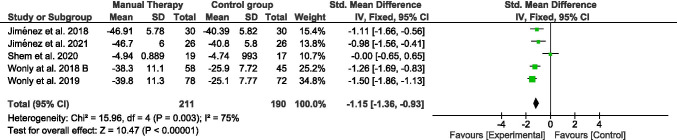
Forest plot of comparison: manual therapy vs control group. Outcome: nerve sensory conduction

Five funnel plots were performed, one for each outcome assessed in this meta- analysis, where changes between manual therapies over the control group were assessed (Figs. 2, 3, 4, 5, 6). In most of them, there seems to appear a good symmetry in the funnel plots; thus, we consider that there is no publication bias. However, for the pain assessment, there seems to be a clear symmetry favoring the studies reporting improvement in this outcome.

## Discussion

The results of this systematic review suggest that conservative treatment based on manual therapy is effective for reducing pain intensity and improve function and nerve conduction studies compared to control or sham in patients CTS.

To our knowledge, this is the first meta-analysis that summarizes manual therapy interventions in patients with CTS. Several systematic reviews have been published including different conservative treatments such as electrotherapy, splinting, therapeutic exercise, or drugs [29–31].

In view of the results, our meta-analysis shows statistical differences between diacutaneous fibrolysis technique to sham or control [15, 26]; glide and tension neurodynamic techniques to sham on symptom function and nerve conduction studies [14, 25, 27]. On the other hand, there were no statistical differences between bone mobilization and neurodynamic techniques; also, the self-myofascial stretching of carpal ligament did not show significant differences on symptoms or function [28].

The results of this meta-analysis are consistent with the previous systematic reviews that showed positive effects after manual therapy treatment on symptoms and function in patients with CTS [21, 29, 31–33]. In these reviews, the intervention included all conservative treatments, whereas in this meta-analysis, the effect of manual therapy interventions in isolation was analyzed.

Diacutaneous fibrolysis effects were analyzed in two studies included in that meta-analysis. They found statistical differences on pain intensity, function, nerve conduction studies, and mechanosensitivity [15, 26]. Several authors have hypothesized the use of soft tissue mobilization around the median nerve to decrease the compression and improve symptoms in patients with CTS [18, 34–37]. Although it has not been studied in depth yet, it seems that the mechanism of diacutaneous fibrolysis could impact on tissue adhesion and increase the connective tissue mobility [38–40]. Thus, as previous authors have suggested, the instrumental soft tissue mobilization of the forearm and wrist could improve the median nerve gliding in the carpal tunnel in patients with CTS [26, 35, 41].

Shem et al. (2020) [28] investigated a self-stretching protocol of carpal tunnel and did not find any difference in any variable. The intervention group’s positive effects did not achieve statistical significance differences compared to the sham group [28]. The self-stretching technique may not be as effective as the intervention applied by the therapist, which may explain the lack of statistically significant results.

Neurodynamic mobilization techniques were applied in three studies included in this meta-analysis. In two of them, the technique was performed by the therapist, based on glide and tension mobilizations. Compared to sham or control groups, more significant results on symptoms, function, and nerve conduction studies were found. Neurodynamic techniques have been proposed to improve the neurophysiological functions of the median nerve and reduce symptoms in patients with CTS [27, 42]. As the median nerve has a lack of longitudinal and transverse excursion, neural mobilizations could restore the normal movement [43]. Our findings are in line with previous authors. Nevertheless, unlike us, they included combined techniques in their treatment protocols, whereas in this meta-analysis, the effects of neurodynamic technique in isolation were analyzed [30, 44, 45].

By contrast, Tal-akabi et al. (2007) did not found differences between neurodynamic and bone mobilization with flexor retinaculum stretch [14]. In this study, the treatment of the interface aimed with musculoskeletal mobilization may positively effect on the neural compression status. In this sense, the comparison between both techniques could not be different in the assessment after one treatment session.

The results observed in this meta-analysis show that the passive intervention based on manual therapy significantly improved pain intensity decrease. This results are in accordance to previous studies that recommend the using conservative treatment to manage symptoms in patients with CTS [4, 12, 29]. A comprehensive model previously proposed could explain the positive effects on pain intensity applying manual therapy, which means that a mechanical force from manual therapy initiates a cascade of neurophysiological responses from the peripheral and central nervous system responsible for the clinical outcomes [19].

BCTS questionnaire is a valid tool to assess symptom severity and function in patients with CTS [46]. All the interventions improved this variable except to self-treatment group. Also, there were no differences between bone carpal mobilizations and neurodynamic techniques.

As previous studies have determined the statistical difference obtained after the interventions included in this meta-analysis, they achieved minimal clinically important difference [47].

Nerve conduction studies are the gold standard for CTS diagnosis to assess the sensory conduction velocity and distal motor latency. The correlation between this variable and symptoms is still not clear [42, 48]. However, nerve conduction studies have potentially great value not only in selecting patients for a specific treatment but also in the objective assessment of treatment efficacy in CTS, especially when they significantly correlate with clinical outcome measures. Neurodynamic mobilizations and diacutaneous fibrolysis techniques obtained statistical significance in nerve conduction studies after treatment. No previous studies providing data on the minimum detectable difference in the values obtained in the neurophysiological parameters were found. The results of this meta-analysis are in accordance to previous studies that applied conservative treatment achieved improvements on nerve conduction studies [49] but differ from others that no showed significant differences [50, 51]. Again, it is important to highlight that the interventions were applied in isolation compared to previous studies that combined many treatments.

Methodological quality analysis showed a high overall quality supporting the results observed in this systematic review. The most shared bias in the studies included was the lack of blinding of the therapist who administered the therapy and the analysis by intention to treat. These aspects are usual in previous reviews of clinical trial involving manual therapy techniques.

There are some limitations of this systematic review and meta-analysis. Therefore, the obtained results should be interpreted with caution. First, as reflected in the statistic heterogeneity study of the meta-analysis, the included studies have shown from moderate to high heterogeneity. Despite the clinical use of manual therapy techniques, the lack of randomized clinical trials leads to pull different techniques under the same concept and thus to increase methodological heterogeneity. Because of technique variability, the number of sessions and the total duration of treatment differ between the studies. Moreover, the dependent variables and the protocol assessment were heterogeneous.

Future research applying manual therapy on patients with CTS is needed in order to support its effectiveness. Moreover, a follow-up may be interesting to analyze if the improvements are maintained in the long-term.

## Conclusion

This study highlights the effectiveness of manual therapy techniques based on soft tissue and neurodynamic mobilizations, in isolation, on pain, physical function, and nerve conduction studies in patients with CTS.

## Supplementary Information

Below is the link to the electronic supplementary material.

Supplementary file1 (DOCX 79 kb)

Supplementary file2 (DOCX 181 kb)

Supplementary file3 (DOCX 136 kb)

## Declarations

This study design and protocol were performed in accordance with the PRISMA Statement. The protocol was registered previously on PROSPERO CRD42020167559.

**PROSPERO registration**: CRD42020167559.
