# Supplementary material for: The effectiveness of manual therapy on pain, physical function, and nerve conduction studies in carpal tunnel syndrome patients: a systematic review and meta-analysis
Source: Int Orthop. 2021 Dec 3;46(2):301–12. doi: 10.1007/s00264-021-05272-2 (PMC8782801; doi:10.1007/s00264-021-05272-2)
Supplement: Supplementary file 1 — Supplementary file1 (DOCX 79 kb) [file 264_2021_5272_MOESM1_ESM.docx]

Appendix 1. Complete Literature Research

**Pubmed Search Formula:**

#1 (“carpal tunnel syndrome”[mh])

#2 ((manual therapy) OR (fibrolysis) OR (diacutaneous) OR (neurodynamic) OR (neural mobilization) OR (neurodinam*) OR (neural tension) OR (massage) OR (manipulative) OR (graston) OR (mobilization) OR (manipulation) NOT (surgical) NOT (surgery) NOT (release))

#3 ((symptom*) OR (functio*) OR (nerve conduction studies) OR (functional capacity) OR (ability) OR (disability) OR (pain))

#4 #1 AND #2 AND #3

Results 70

Data: 27/01/2021

**Web of Science Search Formula:**

#1 (“carpal tunnel syndrome”[mh])

#2 ((manual therapy) OR (fibrolysis) OR (diacutaneous) OR (neurodynamic) OR (neural mobilization) OR (neurodinam*)OR(neural tension) OR(massage) OR (manipulation) OR(mobilization) OR (graston) NOT (surgical) NOT (surgery) NOT (release))

#3 (symptom*) OR (functio*) OR (nerve conduction studies) OR (functional capacity) OR (ability) OR (disability) OR (pain)

#4 #1 AND #2 AND #3

Results: 167

Data: 27/01/2021

**Cochrane Library Search Formula:**

## 1# Carpal tunnel syndrome

## 2# manual therapy OR fibrolysis OR diacutaneous OR neurodynamic OR neural mobilization OR neurodinam* OR neural tension OR massage OR manipulative OR graston OR mobilization OR manipulation NOT surgery OR surgical OR release

## 3# symptom* OR functio* OR nerve conduction studies OR functional capacity OR ability OR disability OR pain

## #4 1# AND 2# AND 3#

Total: 72

Data: 27/01/2021

**SCOPUS Search Formula:**

## #1 ("Carpal tunnel syndrome" )

## 2# ("manual therapy" OR fibrolysis  OR  diacutaneous  OR  neurodynamic  OR  neural  OR

## mobilization OR  neurodinam*  OR  neural  OR tension  OR  massage  OR  manipulative  OR

## graston  OR  mobilization  OR  manipulation  (NOT surgery  OR  surgical  OR  release ))

## 3# (symptom*  OR  functio*  OR  "nerve conduction studies"  OR  "functional capacity"  OR

## ability  OR  disability  OR  pain )

## #4 1# AND 2# AND 3#

Resultados: 255

Data: 27/01/2021

**PEDro Search Formula:**

#1 Carpal Tunnel Syndrome

#2 Stretching, mobilization, manipulation, massage

#3 Clinical trial

## #4 1# AND 2# AND 3#

Results: 57

Date: 27/01/2021

**TRIPDATABASE Search Formula:**

#1 (carpal tunnel syndrome)

#2(title:mobilization OR manipulation OR massage OR neurodynamics OR neural tension OR soft tissue mobilization OR graston)

#3 (control OR placebo)

#4 (functionality OR symptoms)

## #5 1# AND 2# AND 3# AND 5#

Results: 13

Data: 27/01/2021
