# Supplementary material for: The effectiveness of manual therapy on pain, physical function, and nerve conduction studies in carpal tunnel syndrome patients: a systematic review and meta-analysis
Source: Int Orthop. 2021 Dec 3;46(2):301–12. doi: 10.1007/s00264-021-05272-2 (PMC8782801; doi:10.1007/s00264-021-05272-2)
Supplement: Supplementary file 2 — Supplementary file2 (DOCX 181 kb) [file 264_2021_5272_MOESM2_ESM.docx]

Appendix 2. Funnel plots


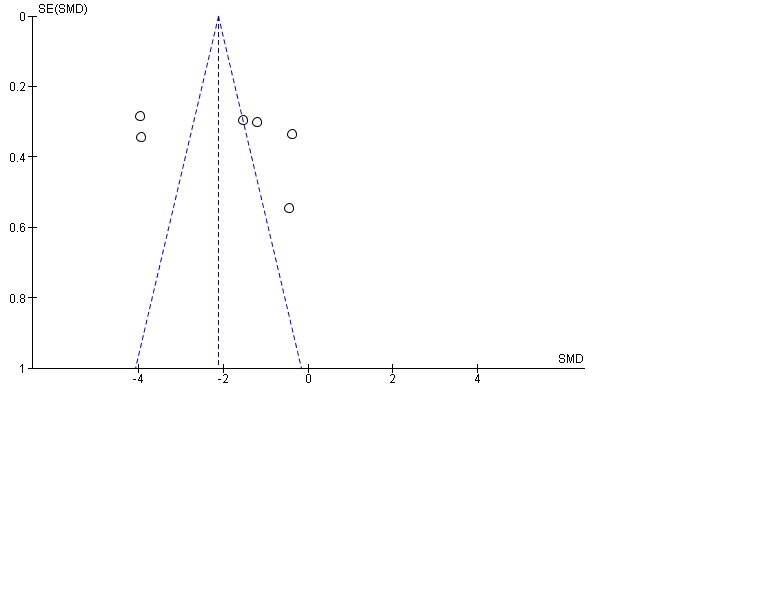
Pain Funnel plot.


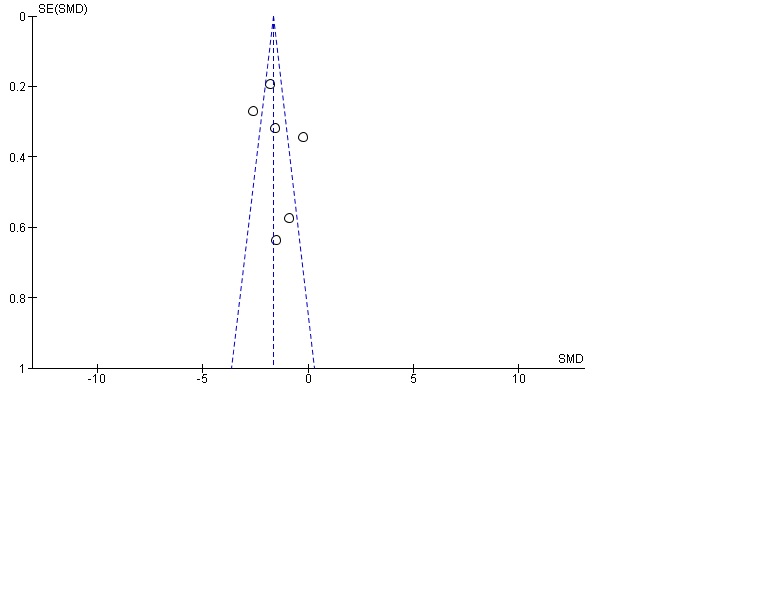


Symptom Severity Scale Funnel plot.


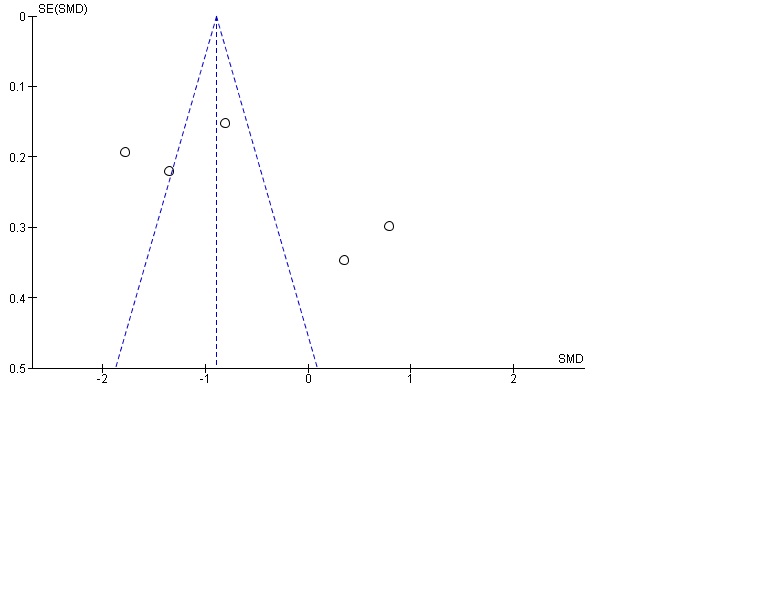


Functional Status Scale Funnel plot.


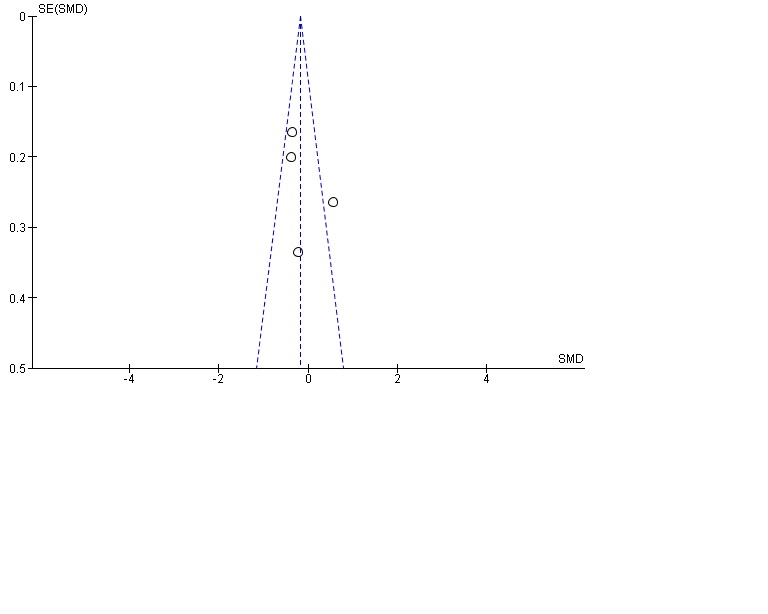


Nerve motor conduction Funnel plot.


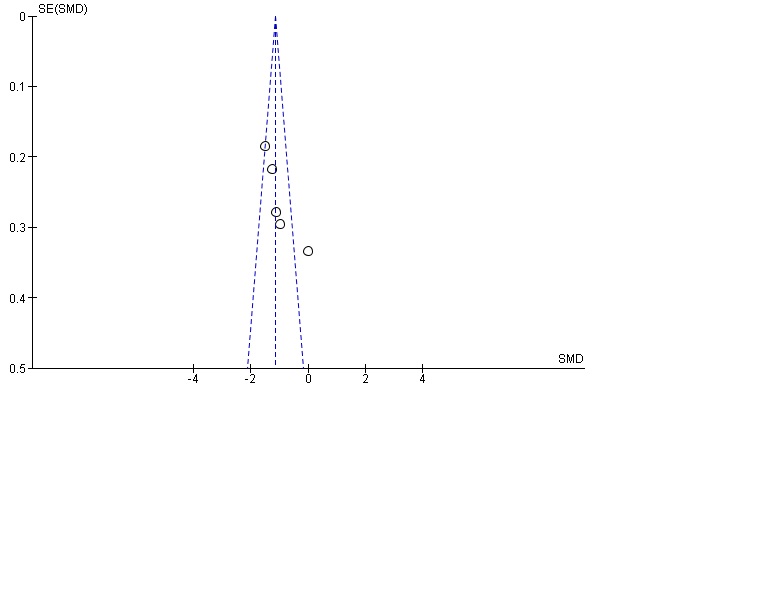


Figure 6. Nerve sensory conduction Funnel plot.
